# Jackknife Model Averaging Prediction Methods for Complex Phenotypes with Gene Expression Levels by Integrating External Pathway Information

**DOI:** 10.1155/2019/2807470

**Published:** 2019-04-08

**Authors:** Xinghao Yu, Lishun Xiao, Ping Zeng, Shuiping Huang

**Affiliations:** ^1^Department of Epidemiology and Biostatistics, School of Public Health, Xuzhou Medical University, Xuzhou, Jiangsu 221004, China; ^2^Center for Medical Statistics and Data Analysis, School of Public Health, Xuzhou Medical University, Xuzhou, Jiangsu 221004, China

## Abstract

**Motivation:**

In the past few years many prediction approaches have been proposed and widely employed in high dimensional genetic data for disease risk evaluation. However, those approaches typically ignore in model fitting the important group structures that naturally exists in genetic data.

**Methods:**

In the present study, we applied a novel model-averaging approach, called jackknife model averaging prediction (JMAP), for high dimensional genetic risk prediction while incorporating pathway information into the model specification. JMAP selects the optimal weights across candidate models by minimizing a cross validation criterion in a jackknife way. Compared with previous approaches, one of the primary features of JMAP is to allow model weights to vary from 0 to 1 but without the limitation that the summation of weights is equal to one. We evaluated the performance of JMAP using extensive simulation studies and compared it with existing methods. We finally applied JMAP to four real cancer datasets that are publicly available from TCGA.

**Results:**

The simulations showed that compared with other existing approaches (e.g., gsslasso), JMAP performed best or is among the best methods across a range of scenarios. For example, among 14 out of 16 simulation settings with PVE = 0.3, JMAP has an average of 0.075 higher prediction accuracy compared with gsslasso. We further found that in the simulation, the model weights for the true candidate models have much smaller chances to be zero compared with those for the null candidate models and are substantially greater in magnitude. In the real data application, JMAP also behaves comparably or better compared with the other methods for continuous phenotypes. For example, for the COAD, CRC, and PAAD datasets, the average gains of predictive accuracy of JMAP are 0.019, 0.064, and 0.052 compared with gsslasso.

**Conclusion:**

The proposed method JMAP is a novel model-averaging approach for high dimensional genetic risk prediction while incorporating external useful group structures into the model specification.

## 1. Introduction

Due to the rapid development of biotechnology [1–4], a large number of high-throughput and low-cost genetic datasets have been generated and provide a broad space to investigate the association between genetic markers and complex diseases/disorders [5–14]. The great success of association studies further promotes the risk prediction and evaluation for complex phenotypes by incorporating into genetic information (e.g., gene expressions or single nucleotide polymorphisms) [15–20]. Due to the high dimensional problem that the number of genetic markers is much larger than the sample size, one of the greatest challenges for genetic risk prediction is that it is difficult to apply traditional statistical methods in large scale molecular omics data. In the past few years, developing prediction methods that can efficiently model high dimensional genetic data has been an active area and attracted much research attention, and a series of novel prediction approaches have been proposed and widely employed for disease risk evaluation or gene expression imputation [21–27]. However, most of those approaches ignore in model fitting the important information of group structures or functional classifications that naturally exist in genetic data. For example, it is well known that genes can be grouped into pathways due to the shared biological function [28]. It has been shown that incorporating such useful group/functional information into model fitting can substantially boost statistical power in genetic association studies and can facilitate our understanding of the genetic architecture of disease variation by heritability partition [27, 29–36]. In genetic data, one of the widely-used group sources is the pathway information in the Kyoto Encyclopedia of Genes and Genomes (KEGG) [37, 38], which integrates information on genomic, chemical, and system functions and groups genes with highly related sequences in terms of the sequence similarity of genes.

Besides being included in genetic association studies and heritability estimation, group/functional information is also recently integrated into genetic risk evaluation with large scale omics data, e.g., the protein-network-based method [39] and the combined-optimal-response-genes (CORGs) approach [40]. Additionally, the regularization methods (e.g., group Lasso) can perform a group selection and estimation by considering the group information [41, 42]. The prediction accuracy can be improved due to the inclusion of grouped functional information [43–45]. For example, Tang et al. [45] recently designed a group spike-and-slab Lasso generalized linear model (gsslasso) that combined KEGG pathway information into model fitting and demonstrated that compared with regularization methods (e.g. Lasso), the average gain of prediction accuracy (measured by area under the curve (AUC)) of gsslasso was about 4.5% for sarcoma, 4.6% for ovarian cancer, and about 1.6% for breast cancer by leveraging gene expression data available from the Cancer Genome Atlas (TCGA) [46].

However, how to appropriately include grouped functional information into genetic prediction models is less understood in the literature. Model-averaging methods [47, 48] offer a natural manner to address this problem by averaging the performance of multiple candidate prediction models which can be efficiently constructed based on grouped genetic datasets. Motivated by this, in the present study, we employ a novel model-averaging approach for high dimensional genetic risk prediction while incorporating KEGG pathway information into the model specification. The proposed model-averaging approach selects the optimal weights across candidate models by minimizing a cross validation criterion in a jackknife way. We thus refer to the method as jackknife model averaging prediction (JMAP). We use extensive simulation studies to evaluate the performance of JMAP and compare it with existing methods. Finally, we apply JMAP to four real cancer datasets that are publicly available from TCGA. To construct candidate prediction models, in the present study, we divide genes in terms of the KEGG pathway information [37, 38].

## 2. Methods and Materials

### 2.1. Overview of the JMAP Method

We first present an overview of JMAP here; the detailed description of JMAP is shown in Supplementary Materials. Briefly, JMAP consists of two-step model fitting procedures: (i) in the first step, we divide the molecular predictors (e.g., genome-wide gene expressions) into *K* biological pathways/groups (e.g., KEGG) and build a series of candidate linear prediction models with gene expression measurements available for various groups; we assume that the pathways are predetermined and that the predictors may overlap across different pathways; (ii) in the second step, we look for a suitable weight vector for averaging across the candidate models to perform a pooled prediction. One of the primary features of JMAP is to allow model weights to vary from 0 to 1 but without the limitation that the summation of weights is necessarily equal to one [47, 48]. As we will see, this weight relaxation is important and critical, resulting in a substantial improvement of the prediction accuracy. JMAP has been implemented within an *R* function freely available at https://github.com/biostatpzeng.

### 2.2. Simulations and Real Data Applications

#### 2.2.1. Simulation Settings

We next carried out extensive simulations to evaluate the prediction performance of JMAP. To make the simulation settings as real as possible, we used gene expression levels obtained from an existing TCGA dataset of breast cancer (see below for further information about this data). For simplicity, we extracted the expression levels for 6,000 randomly selected genes and 500 breast cancer patients and simulated phenotypes using the following model:

(1) $$\begin{array}{c} y=\overset{K}{\underset{j=1}{\sum }}G_{j}\beta_{j}+e,e∼N\left(0,I_{n}\sigma_{e}^{2}\right), \end{array}$$

where *K* is the total number of groups (or pathways); **G**_*j*_ is an *n* × *m*_*j*_ genetic matrix for *m*_*j*_ genes in group *j* with *n* the sample size (here *n*=500), **β**_*j*_ is an *m*_*j*_-dimensional vector of effects sizes; **I**_*n*_ is an *n* × *n* identity matrix; and **e** is an *n*-dimensional vector of independently and normally distributed residuals with variance *σ*_e_^2^. We considered four scenarios with different group partitions. In scenarios 1–3, genes were sequentially divided into 50, 200, or 300 groups with approximately equal genes per each group; no overlapping of genes existed among groups. In scenario 4, we classified genes into 328 groups in terms of the KEGG pathway information (see below for details); note that, under this case, the number of genes included in each group was not equal and ∼21% genes belonged to multiple pathways. Then, following [45], in each scenario, we randomly selected five out of all *K* groups (*K* *=* 50, 200, 300, or 328 as defined above) and generated: (I) the effect sizes **β**_*l*_ (*l* = 1, 2, 3, 4 and 5) in each of the selected groups followed a normal distribution with mean zero and the same variance (say *σ*_*l*_^2^). Under this case, all the genes in the five groups had nonzero effect sizes; (II) unlike case I, here, we assumed that only the genes in the first two groups had nonzero effect sizes and half of the genes in the last three group had nonzero effect sizes; (III) instead of assuming equal proportion of nonzero effect sizes in the last three groups, we set the proportion of nonzero effect sizes to be 80%, 50%, and 20%, respectively; (IV) in this case, we set the proportion of nonzero effect sizes to be 90%, 70%, 50%, 30%, and 10% for the five groups, respectively. The variance parameters *σ*_*l*_^2^ and *σ*_*e*_^2^ were carefully chosen to ensure that **y** had unit variance asymptotically, and the phenotypic variance explained (PVE) by genetic component was 0.3, 0.5, or 0.8 in each case, respectively. The effect sizes for the unselected gene groups were set to zero.

#### 2.2.2. Real Data Applications

We now applied JMAP to four cancer datasets publicly available from TCGA [46], including breast cancer (BRCA), colon and rectal cancer (CRC), colon cancer (COAD), and pancreatic cancer (PAAD). We downloaded both the clinical data and RNAseq gene expression levels for those cancers from UCSC Xena (https://xenabrowser.net/). For each cancer, we first merged the clinical data and gene expression levels measured from primary cancer tissue; then, we removed genes with more than 50% zero expressions and standardized the remaining gene expression levels. The used datasets in this study are summarized in Table 1. Following previous studies [12, 38, 49], for the four cancers, we used the age at initial pathologic diagnosis (i.e., onset age) as phenotypes because the age of onset is an important indicator that the cancer is likely more commonly genetic in origin. We quantile-normalized onset age to a standard normal distribution before prediction analysis.

### 2.3. Model Comparison and Implementation

For the simulated data, the genes were divided into 50, 200, 300, or 328 groups under various scenarios as mentioned before. For the real datasets, we mapped the genes to KEGG pathways by *R* package clusterProfiler (version 3.8.1) after matching gene symbols to Entrez ids [50] and divided the genes into 328 pathway groups. For both simulated and real datasets, following [24], we performed 100 Monte Carlo cross validation (MCCV) data splits by randomly selecting 80% samples as training data and the remaining 20% as test data. We fitted the prediction models in the training data and evaluated the performance in the test data with correlation coefficient (*R*).

As gsslasso was proved to perform better than sparse group Lasso [45]; our competing methods only included Lasso [51], elastic net (ENET) [52], random forest [53], and gsslasso [45]. For both Lasso and ENET, we implemented them via the *R* package glmnet (version 2.0-16), selected the optimal penalty parameters in Lasso and ENET using 100-fold cross validation, and set *α* = 0.50 in ENET as done in [54]. For random forest, we implemented it via the *R* package randomForest (version 4.6-14). For gsslasso, we implemented it via the *R* package BhGLM (version 1.1.0). Following [45], we selected the optimal penalty parameter of gsslasso by setting the slab scale (denoted by *s*_1_) to 1, calculated the accuracy of prediction for a series values for the spike scale (denoted by *s*_0_) (i.e., *s*_0_ = 0.01 × *m*, *m* = 0.1, 1, 2,…, 9), and chose the optimal value for *s*_0_ that resulted in a highest prediction. We solved the quadratic problem in JMAP (Equations (7) and (8) in Supplementary Materials) using the optim function in *R* statistical software. We further contrasted the prediction performance of all other methods with that of JMAP by taking the difference of *R* between the other methods and JMAP. Therefore, an *R* difference below zero suggests worse performance than JMAP.

## 3. Results

### 3.1. Results of the Simulation Studies

**Figure 1:**
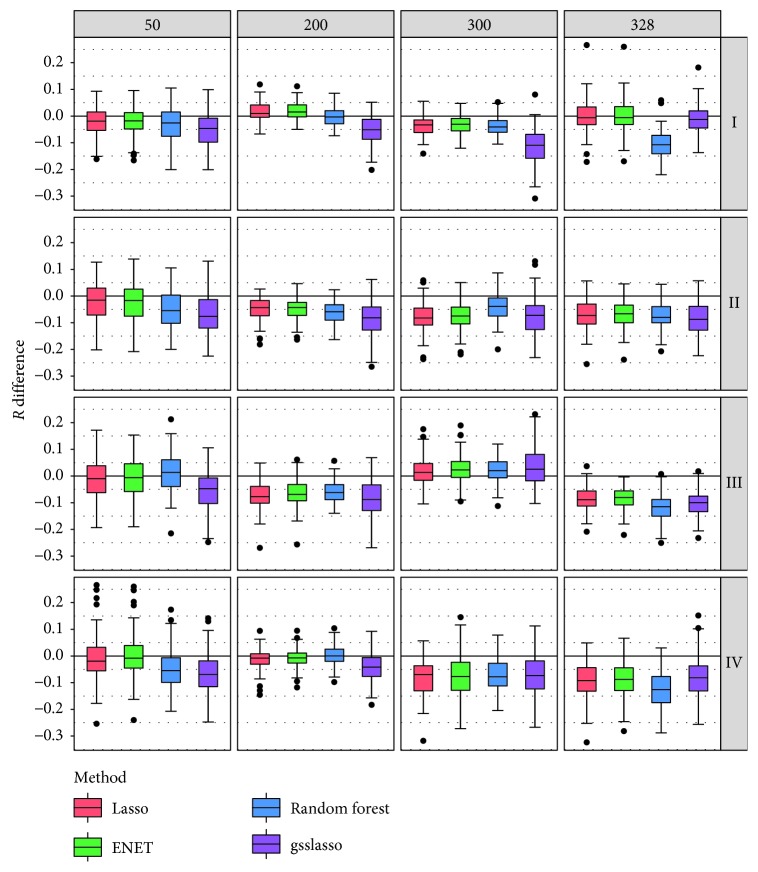
Comparison of predictive performance of four models with JMAP with PVE = 0.3. Performance is measured by *R* difference with respect to JMAP; therefore, a negative value (i.e., values below the horizontal line) indicates worse performance than JMAP. In each setting, five groups with nonzero effect sizes were selected; I represents the settings where all the genes in the five groups had nonzero effect sizes; II represents the settings where only the genes in the first two groups had nonzero effect sizes, and half of the genes in the last three groups had nonzero effect sizes; III represents the settings where the effect sizes of the first two groups were nonzero, and the proportion of nonzero effect sizes in the last three groups was 80%, 50%, or 20%; IV represents the settings where the proportion of nonzero effect sizes in the five groups was 90%, 70%, 50%, 30%, or 10%. The predictive performance was assessed across 100 replicates in each scenario.

The simulation results for the difference of *R* with PVE = 0.3 are shown in Figure 1 with the original *R* values shown in Figure S1. There are 16 combinations presented in Figure 1. Compared with other existing approaches (i.e., Lasso, ENET, random forest, and gsslasso), we find that, except two situations, JMAP performed best or is among the best methods in most of the combinations (14 out of 16). For example, among those 14 settings, JMAP has an average of 0.075 higher prediction accuracy compared with gsslasso, with the difference of *R* ranging from 0.023 to 0.116. In the setting with 200 groups in scenario I (where all the genes in the five groups had nonzero effect sizes), JMAP is better than gsslasso (0.056 higher) and is comparable with random forest, while it behaves slightly worse than Lasso (0.012 lower) and ENET (0.013 lower). In the setting with 300 group in scenario III (where the genes among the first two groups had nonzero effect sizes, but some of the genes in the rest three groups are null with various null proportions), all the four competitive methods (i.e., Lasso, ENET, random forest, and gsslasso) have a higher prediction accuracy relative to JMAP. The simulation results for PVE = 0.5 and 0.8 are displayed in Figures S2–S5 in Supplementary Materials; we observed the similar pattern that JMAP performs better or is as good as other competing methods in most of the simulated settings. We further checked the estimated weights for the candidate models in all the scenarios and found that the weights for the true candidate models (i.e., those with nonzero effect sizes) have much smaller chances to be zero compared with those for the null candidate models and are substantially greater in magnitude (Table S1).

### 3.2. Results of the Real Data Applications

**Figure 2:**
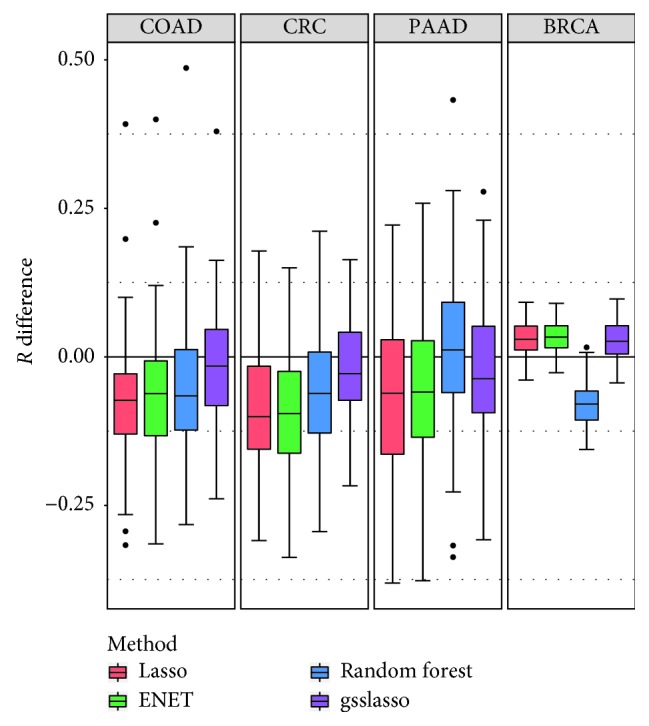
Comparison of predictive performance of four models with JMAP for the four phenotypes from the TCGA datasets. Performance is measured by *R* difference with respect to JMAP; therefore, a negative value (i.e., values below the horizontal line) indicates worse performance than JMAP. The predictive performance was assessed across 100 MCCV replicates. BRCA: breast cancer; CRC: colon and rectal cancer; COAD: colon cancer; PAAD: pancreatic cancer.

Now, we turn to the real application of the TCGA data (Table 1). The results of *R* differences of other four methods compared with JMAP are presented in Figure 2. Totally, JMAP performs comparably or better compared with the other methods. For example, for the COAD, CRC, and PAAD datasets, JMAP has the highest predictive power, followed by gsslasso. Compared with gsslasso, in these three datasets, the gains of predictive accuracy of JMAP are 0.019, 0.064, and 0.052, respectively. In the PAAD dataset, JMAP is better than Lasso, gsslasso, and ENET, while random forest has the highest prediction accuracy. In the BRCA dataset, except for random forest, the rest of the methods (i.e., Lasso, gsslasso, and ENET) have a higher prediction accuracy compared with JMAP.

## 4. Discussion

In the present study, we have employed a novel statistical method, JMAP, for genetic prediction and evaluation of complex phenotypes from the publicly available TCGA datasets. Traditionally, the classical model-averaging methods first build a series of candidate models with various degrees of model complexity; then combine all the candidate models together to boost the prediction performance by specifying greater weights onto better models; and require the summation of the model weights is equal to one [47, 55, 56]. However, unlike those previous methods, JMAP relaxes the constraint of summing the weights of candidate models up to one. By removing this restriction and including genetic pathway information, as we have demonstrated in the simulations and real data applications, JMAP has shown higher prediction accuracy compared with existing approaches. Furthermore, it is natural to examine whether the weight restriction can be further relaxed to allow them to vary between −1 and 1 [57]. However, we found that this further relaxing may be not beneficial for improving the prediction performance, leading to low accuracy of genetic prediction (Figure S7). Additionally, because each candidate model is fitted with ordinary least squares method which leads to an analytical solution for the effect sizes and because the weight estimation is optimized through a constrained quadratic manner, JMAP is thus computationally efficient and can be easily scalable to the high dimensional genetic risk prediction problem. For example, in the real data applications, it takes only about 3, 3, 110, 15, and 18 seconds on average for Lasso, ENET, random forest, gsslasso, JMAP on the COAD datasets, respectively.

In practice, the candidate models for model averaging are typically established in terms of prior knowledge or expert viewpoints, and the number of the candidate models (i.e., *K* in our study) is assumed to be uncertain. To address this problem, Ando and Li [48] recently proposed first to partition predictors (equivalent to genes in our study) based on the marginal correlation magnitude between each predictor and the response and then adaptively prepared for candidate model for each partition. This strategy is a flexible way and avoids the requirement of external information, while it may be suboptimal if there is informative prior information that can be utilized. In contrast, in our study, we explicitly preassigned the number of candidate models for JMAP. Indeed, using simulations, we have discovered that JMAP possessed consistently good prediction performance across various candidate model partitions. In our real data applications, we also directly built the candidate models for JMAP based on useful KEGG pathway information which characterizes the biological functions for various sets of genes [37, 38] and can result in each candidate model having unique strength in capturing certain aspects of prediction ability. Applying external informative pathways to establish candidate models in JAMP can lead to at least three benefits: (i) it does not need to search for the appropriate number of candidate models by partitioning all the genes; thus, it is computationally faster; (ii) relying on previously well-validated pathway information, the established candidate models are more biologically meaningful; (iii) finally, the marginal correlation way typically groups a given gene into only one candidate model [48], while in practice, a gene often can be involved in multiple pathways and will be thus included into several candidate models, e.g., in our analysis, about 21% genes can be grouped into at least two pathways. More generally, under the context of model averaging, JAMP can naturally handle the overlapping group structures—a phenomenon that is frequently encountered in pathway-based data analyses [58]. It has been shown that efficiently incorporating the overlapping group structures into model fitting can raise the prediction performance [45]. Hence, JAMP has the potential for further enhancing prediction accuracy. Figures S8 and S9 show the predictive performance of JMAP and MCV2 (i.e., the model-averaging method described in [48], where the candidate models are constructed based on the marginal correlation magnitude between each predictor and the response) for phenotypes from both the simulated and real-life datasets and illustrate the advantage of preassigning the candidate models.

As mentioned before, the greatest feature of JMAP is that the sum of the model weights is equal to one is relaxed. In contrast, the traditional model-averaging approaches often assume that candidate models are equally competitive and thus assign equal weights for all the candidate models. However, in practice, this does not necessarily hold given the fact that only a few pathways are active and the other pathways may have a small or ignorable influence on complex phenotypes. Furthermore, as shown in the simulations and real data applications, relaxing the weights limitation in JMAP allows to put more weights on candidate models that were constructed for possibly active pathways, potentially increasing the prediction performance. Theoretically, the benefit of relaxing the weights limitation in model-averaging approaches has been proved in [48].

It is worth noting that in the candidate model of JMAP, the least squares estimate in Equation (2) (Supplementary Materials) is ill-conditional when the number of genetic markers is larger than the sample size for some genes. For example, in our analysis, there are 5.5% and 5.2% pathways with the number of genes greater than the sample sizes for the PAAD and COAD datasets, respectively. Under this situation, regularization methods (e.g., Lasso) can be applied to each candidate model [59]; however, doing this can lead to substantial increase in computational time because the simple closed-form solution cannot be available for candidate model. In the present study, by borrowing the idea of ridge regression [60, 61], we have attempted to add a nonnegative constant *δ* into the estimates, i.e., replacing **G**_*j*_^*T*^ **G**_*j*_ with **G**_*j*_^*T*^ **G**_*j*_ *+δ* **I**_*m*_*j*__ (Equation (2) in the Supplementary Materials). In our paper, we primarily set *δ* to be one and found that JMAP is robust with regard to various values of *δ* with simulations (Figure S10). We emphasize that this is an ad hoc modification which has no clear theoretical foundation. Further investigation of JMAP under the context that the dimension of candidate model is larger than the sample size is an important and interesting topic and is our next research direction.

Finally, the current version of JMAP described in our study is constructed only for continuous phenotypes. Extending model averaging from linear to nonlinear regression under the high dimensional situations was recently investigated [57]. However, although not mentioned, an explicit model assumption in their study is that the number of the predictors in each candidate generalized linear model should be much less than the sample size to ensure the estimates can be identifiable. Therefore, their methods cannot be applied to our case where the number of the genes for some candidate models is easy to be greater than the sample size as mentioned before. Thus, in our real data application, we had to directly fit linear candidate models for binary phenotypes by treating them as continuous values following previous studies [21–23, 25]. Theoretically, modeling binary data with linear models can be justified by the fact that the linear model can be viewed as a first order Taylor approximation to the generalized linear model, and this approximation is accurate when the effect size is weak and small [21]—a condition which generally satisfies because it has been shown that most complex phenotypes are polygenic and are influenced by many genetic variants with small effect sizes [7]. Nevertheless, extending the JMAP model for application to noncontinuous phenotypes in high dimensional prediction problems warrants more explorations.

## Figures and Tables

**Table 1:** Sample sizes and the number of genes for each cancer in the TCGA dataset used in our analysis.

Phenotypes	Initial gene expression data		Initial clinical data (*N*)	Final data after quality control	
	*N*	*G*		*N*	*G*
BRCA	1,218	20,531	1,247	1,083	17,675
COAD	329	20,531	551	275	17,493
CRC	434	20,531	736	367	17,510
PAAD	183	20,531	196	178	17,675

*Note. N* is the sample size and *G* denotes the number of genes. The average number of genes incorporated in each pathway for the seven phenotypes was 65 (ranging from 1 to 1,139), and about 21% genes belonged to multiple pathways. BRCA: breast cancer; CRC: colon and rectal cancer; COAD: colon cancer; PAAD: pancreatic cancer.

## Data Availability

The TCGA data are publicly available from https://xenabrowser.net/. The BhGLM software is available from http://github.com/nyiuab/BhGLM. The glmnet package is available from https://cran.r-project.org/web/packages/glmnet/index.html. Random forest software is available from https://cran.r-project.org/web/packages/randomForest/index.html.

## Supplementary Materials

Supplementary Materials A detailed description for the proposed JMAP approach. Briefly, JMAP is a novel model-averaging based genetic risk prediction approach that can incorporate the group biological information of genetic alterations into prediction modeling. It consists of two-step model fitting procedures: (1) construct candidate models and (2) optimize the model weights.
