# Supplementary material for: Jackknife Model Averaging Prediction Methods for Complex Phenotypes with Gene Expression Levels by Integrating External Pathway Information
Source: Comput Math Methods Med. 2019 Apr 8;2019:2807470. doi: 10.1155/2019/2807470 (PMC6476151; doi:10.1155/2019/2807470)
Supplement: Supplementary Materials — A detailed description for the proposed JMAP approach. Briefly, JMAP is a novel model-averaging based genetic risk prediction approach that can incorporate the group biological information of genetic alterations into prediction modeling. It consists of two-step model fitting procedures: (1) construct candidate models and (2) optimize the model weights. [file 2807470.f1.doc]

**Supplementary Text for “Jackknife model averaging prediction methods for complex phenotypes with gene expression levels by integrating external pathway information”**

Xinghao Yu^1^, Lishun Xiao^1^, Ping Zeng^1*^, Shuiping Huang^1*^

^1^Department of Epidemiology and Biostatistics, School of Public Health, Xuzhou Medical University, Xuzhou, Jiangsu, 221004, People's Republic of China

* Correspondence: zpstat@xzhmu.edu.cn; [hsp@xzhmu.edu.cn](mailto:hsp@xzhmu.edu.cn)

#### The description of JMAP

We here provide a detailed description for the proposed JMAP approach. Briefly, JMAP is a novel model-averaging based genetic risk prediction approach that can incorporate the group biological information of genetic alterations into prediction modeling . It consists of two-step model fitting procedures.

#### Construct candidate models

Let **G***_j_* be the genetic matrix for a set of predictors (e.g. expression levels for genes) in group *j* (*j* = 1, 2, …, *K*; where *K* is the total number of groups that are assumed to be known and preassigned in terms of prior knowledge; we discuss the case where *K* is unknown in the Discussion section in the main text) and **y** be a continuous phenotype. Note that the number of predictors (denoted by *m_j_*) for each **G***_j_* is not necessarily identical. Suppose both **G***_j_* and **y** are standardized to have zero mean and variance one.

We denote a set of candidate models by *C*_1_, *C*_2_, …, *C_K_*, and employ the following linear model to link the relationship between **G***_j_* and **y**

$C_{j}: y \text{=} G_{j}\beta_{j} + e, e \sim N(0, \sigma_{j}^{2}I_{n})$,

where the intercept term is ignored as the phenotype **y** is assumed to be standardized; ***β****_j_* is an *m_j_*-dimensional vector of effects sizes for the predictors; **I***_n_* is an *n*×*n* identity matrix with *n* the sample size; and ***e***is an *n*-dimensional vector of independently and normally distributed residuals with variance $\sigma_{j}^{2}$. Then, the least squares method is used to estimate ***β****_j_* for the candidate model *C_j_*

$\hat{\beta}_{j} = \mathrm{argmin}\{\frac{1}{2}(y - G_{j}\beta_{j})^{T}(y - G_{j}\beta_{j})\} = (G_{j}^{T}G_{j})^{-1}G_{j}^{T}y$.

The least squares prediction is naturally estimated by $\hat{\mu}_{j}=G_{j}\hat{\beta}_{j}$.

#### Optimize the model weights

The basic idea of model averaging is to combine all the candidate models with appropriate model weights, which play a key role in the prediction performance of the final pooled model. After partitioning genes into *K* groups by group information and building the candidate model *C_j_*, we next calculate the optimal weights for these models. Denote the hat matrix $G_{j}(G_{j}^{T}G_{j})^{-1}G_{j}^{T}$ by **H***_j_* for model *C_j_*, and the *K*-dimensional weight vector by **w** = (*w*_1_, *w* _2_, …, *w_K_*)*^T^* which comes from the unit hypercube: *Q_n_*={**w**∈[0,1]*^K^*: 0≤*w_j_*≤1}. The final model average prediction is estimated as

$$\hat{\mu}_{w} = \sum_{j=1}^{K} w_{j}\hat{\mu}_{j} = \sum_{j=1}^{K} w_{j}G_{j}(G_{j}^{T}G_{j})^{-1}G_{j}^{T}y= \sum_{j=1}^{K} w_{j}H_{j}y= H_{w}y,$$

where $H_{w}=\sum_{j=1}^{K} w_{j}H_{j}$. Then we estimate the weights **w** using the jackknife approach (also referred to as leave-one-out cross-validation) . In the jackknife method, the predicted value for the *i*th individual is calculated in terms of the candidate model that is constructed by removing this individual

$\tilde{\mu}_{j}^{i}=G_{j}^{i}((G_{j}^{(-i)})^{T}G_{j}^{(-i)})^{-1}(G_{j}^{(-i)})^{T}y^{(-i)}$,

here $G_{j}^{i}$ is the predictor vector for individual *i* in model *C_j_*, $G_{j}^{(-i)}$ and $y^{(-i)}$ denote **G***_j_* and **y** with the *i*th row deleted, respectively. Let $\tilde{\mu}_{j} = (\tilde{\mu}_{j}^{1}, \tilde{\mu}_{j}^{2}, ..., \tilde{\mu}_{j}^{n})$ be the vector of the predicted value for all individuals in model *C_j_*. Under the framework of the linear jackknife model we can obtain the following simple relationship

$$\tilde{\mu}_{j} = \tilde{H}_{j}y\text{,} \tilde{H}_{j} = D_{j}(H_{j}-I_{n})+I_{n}\text{,}$$

where **D***_j_* is an *n*×*n* diagonal matrix with the *i*th diagonal element equal to $(1-h_{j}^{i})^{-1}$ and $h_{j}^{i}$ is the *i*th diagonal element of **H***_j_*. Thus, the jackknife predicted value can be estimated as

$\tilde{\mu}_{w} = \sum_{j=1}^{K} w_{j}\tilde{\mu}_{j} = \sum_{j=1}^{K} w_{j}\tilde{H}_{j}y = \tilde{H}_{w}y$,

where $\tilde{H}_{w} = \sum_{j=1}^{K} w_{j}\tilde{H}_{j}$. Then, we next use the sum of squared residuals of the jackknife predicted value to set up the cross-validation (CV) criterion

$$\begin{matrix} \text{CV}_{w}=(y-\tilde{H}_{w}y)^{T}(y-\tilde{H}_{w}y), \\ =\mathbf{y}^{T}\mathbf{y}-2\sum_{j=1}^{K} w_{j}\mathbf{y}^{T}\tilde{H}_{j}\mathbf{y}+\sum_{j=1}^{K} \sum_{m=1}^{K} w_{j}w_{m}\mathbf{y}^{T}(\tilde{H}_{j})^{T}\tilde{H}_{m}\mathbf{y}, \\ =\mathbf{y}^{T}\mathbf{y}-2w^{T}x+w^{T}Zw, \end{matrix}$$

where **x** is a *K*-dimensional vector with the *j*th element $x_{j}=\mathbf{y}^{T}\tilde{H}_{j}\mathbf{y}$, and **Z** is a *K*×*K* dimensional matrix with (*j*, *m*)th element $Z_{j, m}=\mathbf{y}^{T}(\tilde{H}_{j})^{T}\tilde{H}_{m}\mathbf{y}$. The optimal weight vector is obtained by minimizing CV**_w_**

$\hat{w}=\mathrm{argmin}_{w\in Q_{n}}\text{CV}_{w}, \text{subject} \text{to} 0\leq w_{j}\leq1$.

In the present paper, the optimization in Equation (8) is implemented by using the optim function in R software. Again, note that the summation of weight vector limited to one is not necessary in this study. Finally, the phenotypic prediction estimate based on a new set of predictors (say $G_{j}^{\text{new}}$) can be easily obtained as

$\hat{\mu}=\sum_{j=1}^{K} \hat{\mu}_{j}^{\text{new}}\hat{w}_{j}=\sum_{j=1}^{K} G_{j}^{\text{new}}\hat{\beta}_{j}\hat{w}_{j}$,

where $G_{j}^{\text{new}}$ has the same partition as **G***_j_* in terms of prior knowledge and $\hat{\beta}_{j}$ is given in Equation .

### Supplementary Figures and Tables


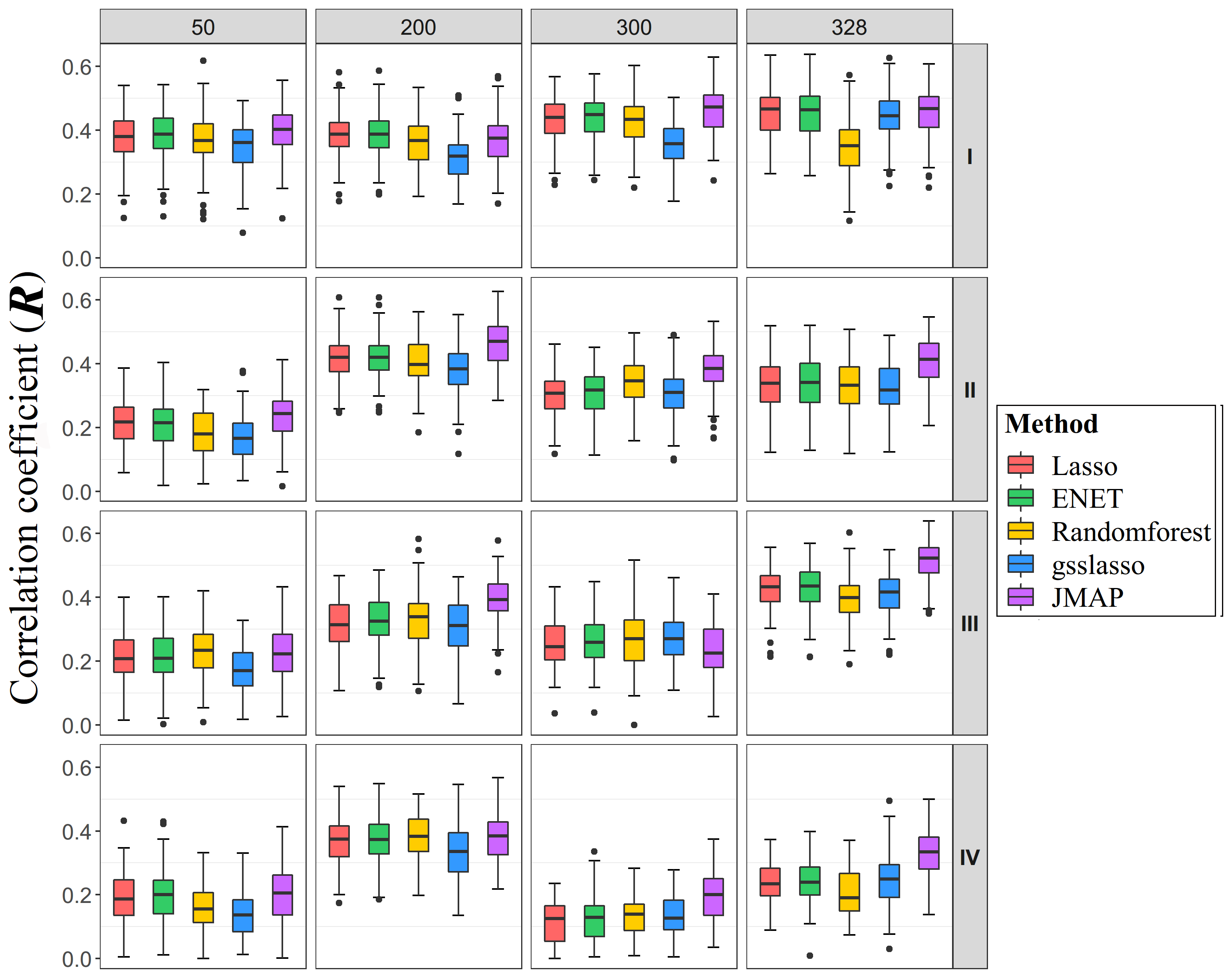


**Fig. S1 Comparison of predictive performance of four models with JMAP with PVE=0.3.** Performance is measured by *R*; In each setting, five groups with non-zero effect sizes were selected; I represents the settings where all the genes in the five groups had non-zero effect sizes; II represents the settings where only the genes in the first two groups had non-zero effect sizes and half of the genes in the last three group had non-zero effect sizes; III represents the settings where the effect sizes of the first two groups were non-zero and the proportion of non-zero effect sizes in the last three groups was 80%, 50% or 20%, respectively; IV represents the settings where the proportion of non-zero effect sizes in the five groups was 90%, 70%, 50%, 30% or 10%.The predictive performance was assessed across 100 replicates in each scenario.


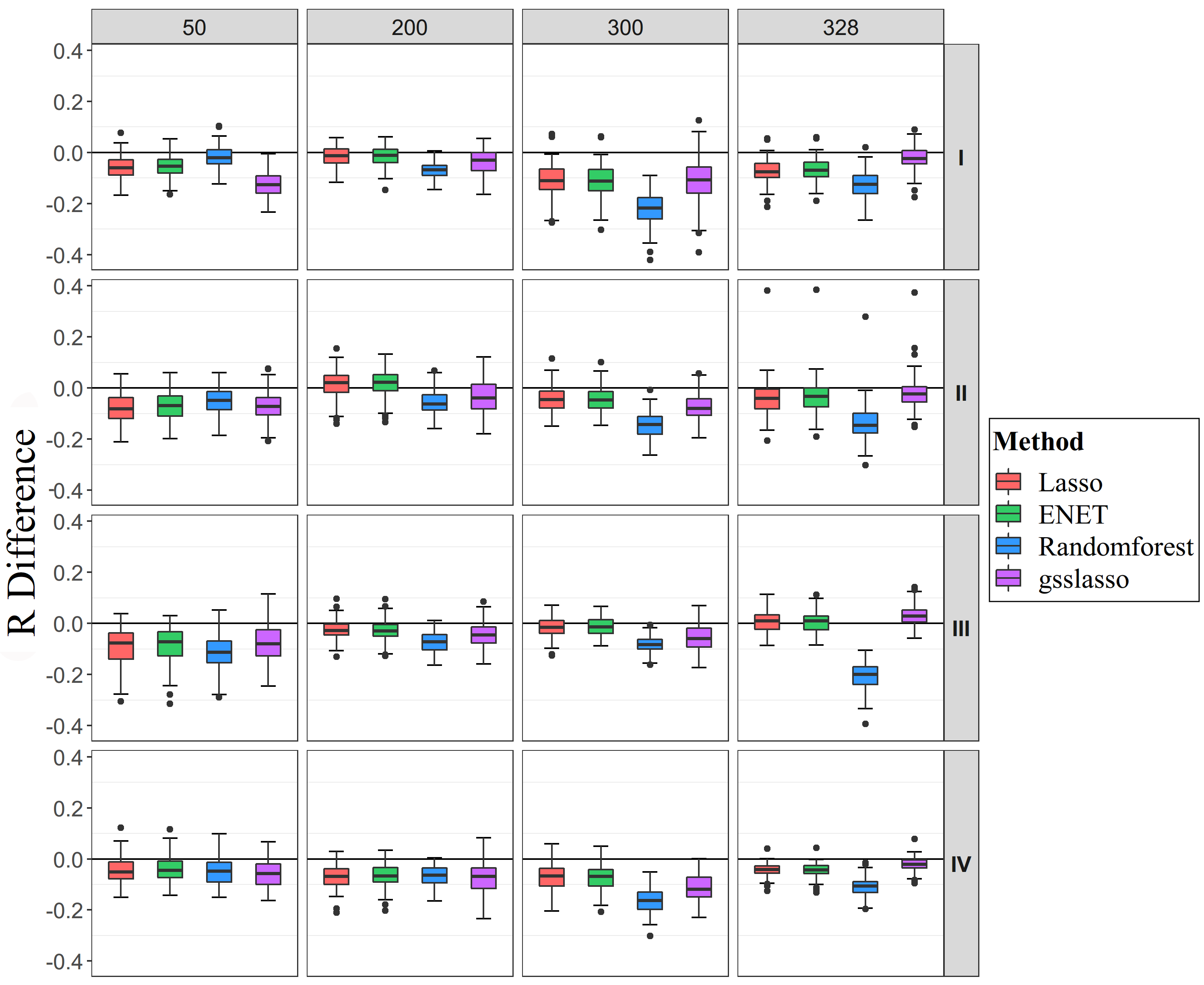


**Fig. S2 Comparison of predictive performance of four models with JMAP with PVE=0.5.** Performance is measured by *R* difference with respect to JMAP; therefore, a negative value (i.e., values below the horizontal line) indicates worse performance than JMAP. In each setting, five groups with non-zero effect sizes were selected; I represents the settings where all the genes in the five groups had non-zero effect sizes; II represents the settings where only the genes in the first two groups had non-zero effect sizes and half of the genes in the last three group had non-zero effect sizes; III represents the settings where the effect sizes of the first two groups were non-zero and the proportion of non-zero effect sizes in the last three groups was 80%, 50% or 20%; IV represents the settings where the proportion of non-zero effect sizes in the five groups was 90%, 70%, 50%, 30% or 10%. The predictive performance was assessed across 100 replicates in each scenario. Among all the settings, JMAP has an average of 0.055 higher prediction accuracy compared with gsslasso, with the difference of *R* ranging from -0.041 to 0.122.


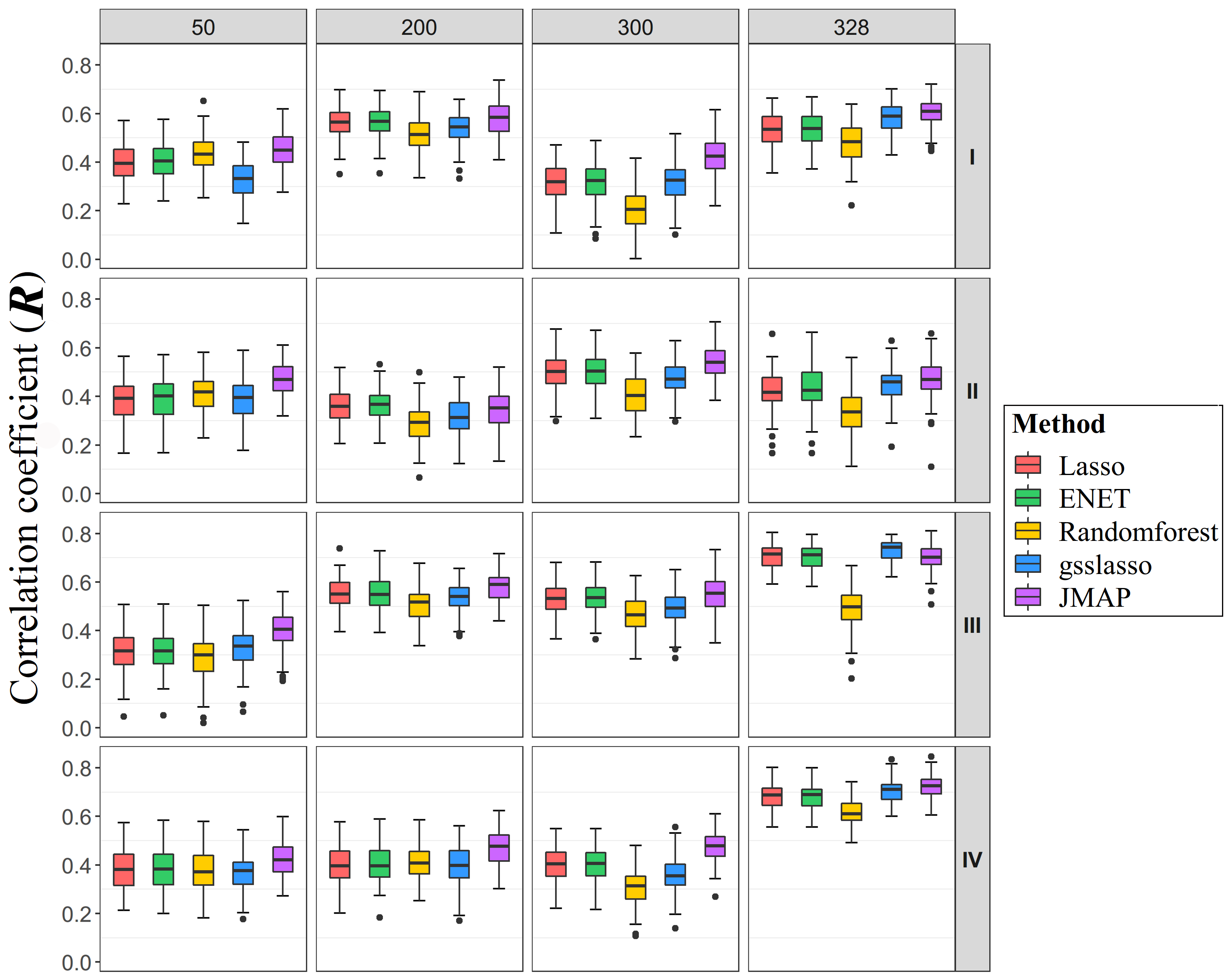


**Fig. S3 Comparison of predictive performance of four models with JMAP with PVE=0.5.** Performance is measured by *R*; In each setting, five groups with non-zero effect sizes were selected; I represents the settings where all the genes in the five groups had non-zero effect sizes; II represents the settings where only the genes in the first two groups had non-zero effect sizes and half of the genes in the last three group had non-zero effect sizes; III represents the settings where the effect sizes of the first two groups were non-zero and the proportion of non-zero effect sizes in the last three groups was 80%, 50% or 20%, respectively; IV represents the settings where the proportion of non-zero effect sizes in the five groups was 90%, 70%, 50%, 30% or 10%. The predictive performance was assessed across 100 replicates in each scenario.


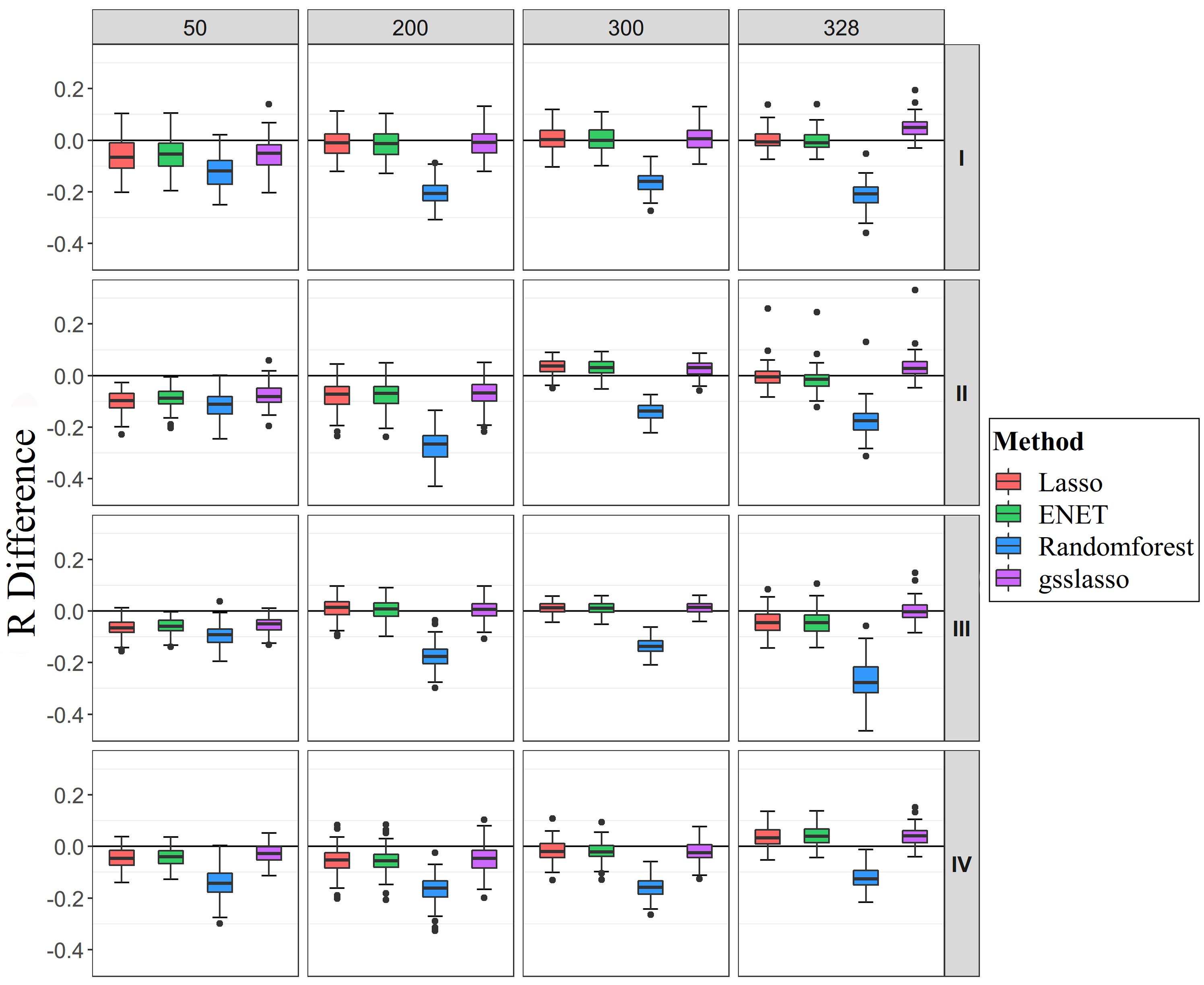


**Fig. S4 Comparison of predictive performance of four models with JMAP with PVE=0.8.** Performance is measured by *R* difference with respect to JMAP; therefore, a negative value (i.e., values below the horizontal line) indicates worse performance than JMAP. In each setting, five groups with non-zero effect sizes were selected; I represents the settings where all the genes in the five groups had non-zero effect sizes; II represents the settings where only the genes in the first two groups had non-zero effect sizes and half of the genes in the last three group had non-zero effect sizes; III represents the settings where the effect sizes of the first two groups were non-zero and the proportion of non-zero effect sizes in the last three groups was 80%, 50% or 20%; IV represents the settings where the proportion of non-zero effect sizes in the five groups was 90%, 70%, 50%, 30% or 10%. The predictive performance was assessed across 100 replicates in each scenario. Among all the settings, JMAP has an average of 0.013 higher prediction accuracy compared with gsslasso, with the difference of *R* ranging from -0.055 to 0.072.


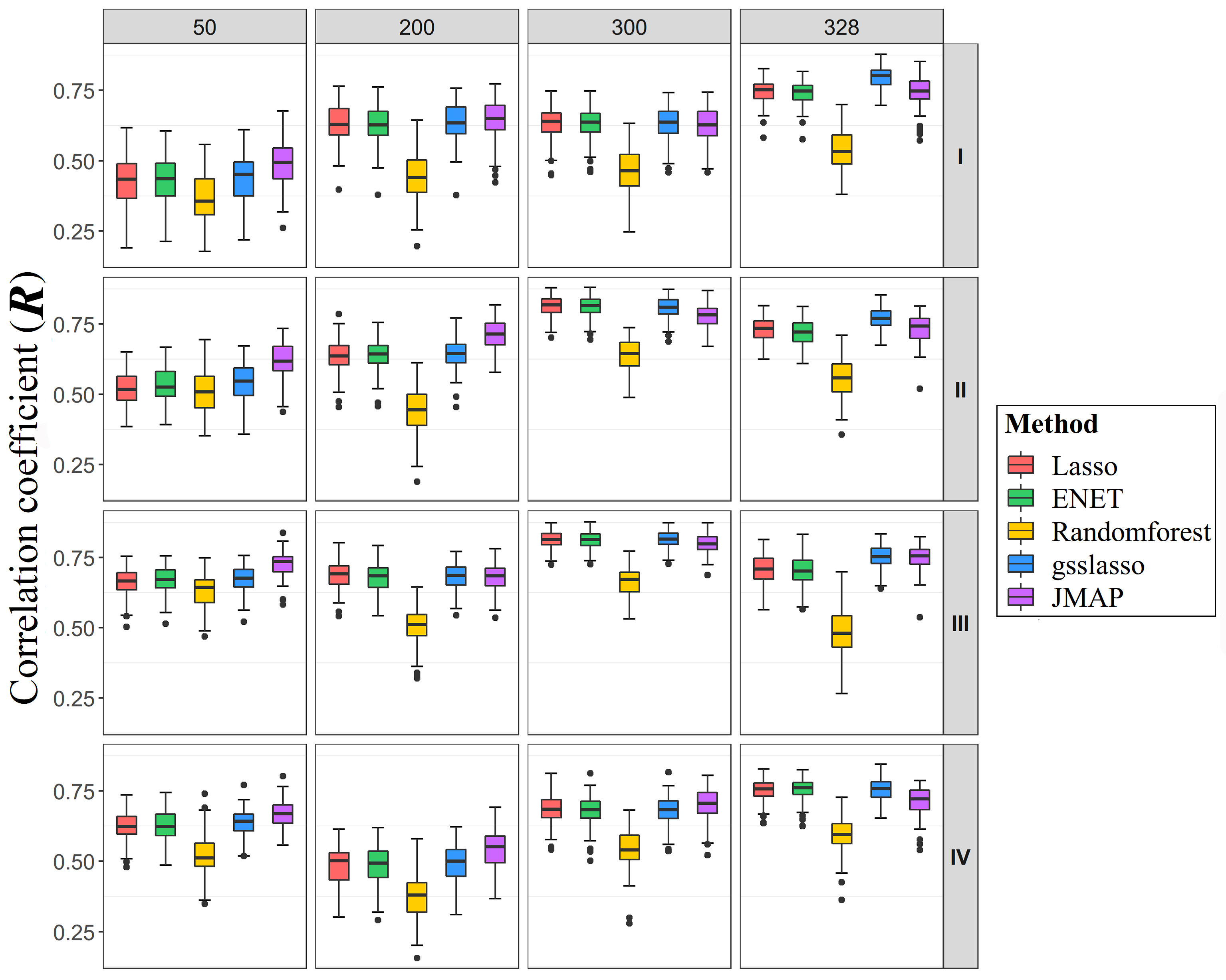


**Fig. S5 Comparison of predictive performance of four models with JMAP with PVE=0.8.** Performance is measured by *R*; In each setting, five groups with non-zero effect sizes were selected; I represents the settings where all the genes in the five groups had non-zero effect sizes; II represents the settings where only the genes in the first two groups had non-zero effect sizes and half of the genes in the last three group had non-zero effect sizes; III represents the settings where the effect sizes of the first two groups were non-zero and the proportion of non-zero effect sizes in the last three groups was 80%, 50% or 20%, respectively; IV represents the settings where the proportion of non-zero effect sizes in the five groups was 90%, 70%, 50%, 30% or 10%.The predictive performance was assessed across 100 replicates in each scenario.


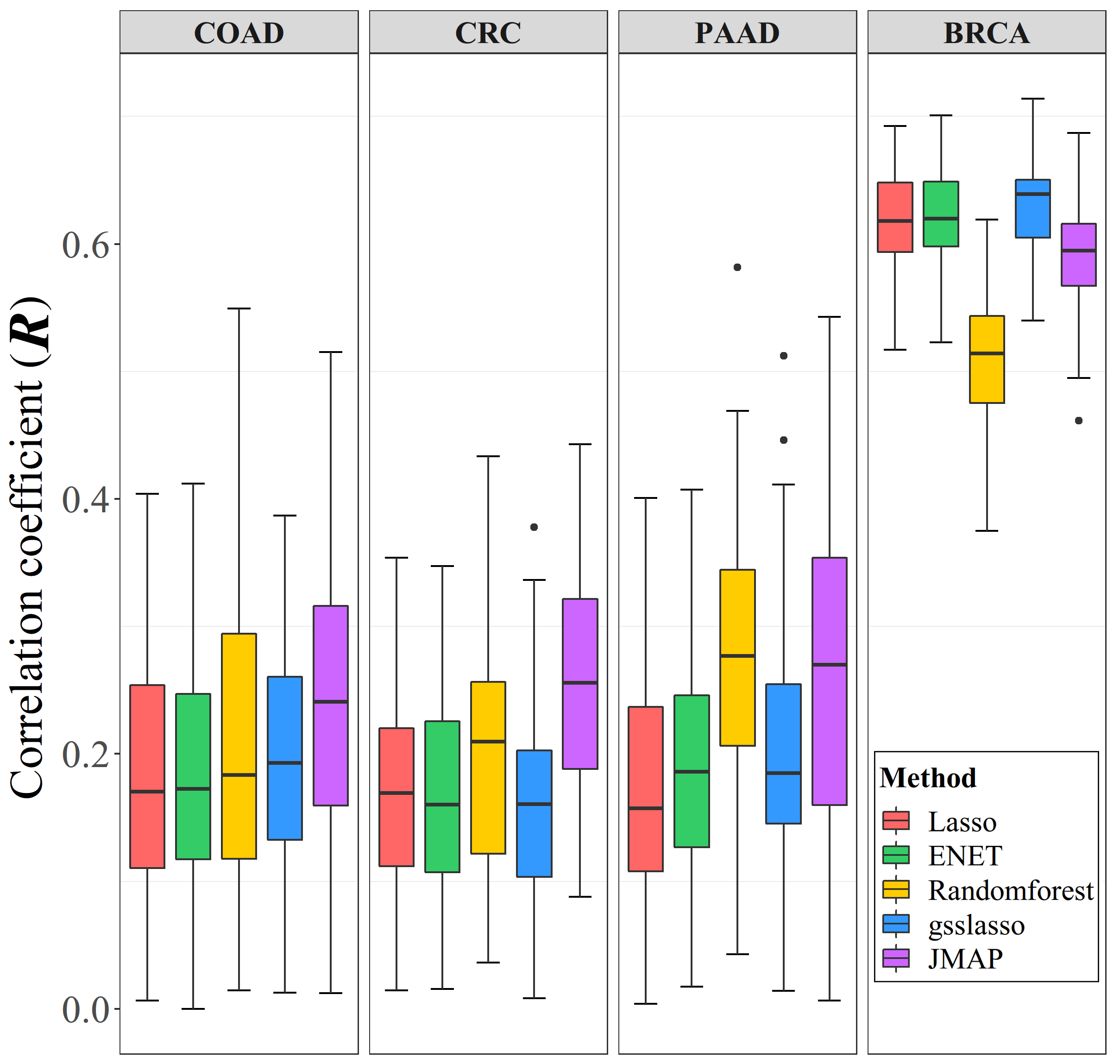


**Fig. S6 Comparison of predictive performance of four models with JMAP for four phenotypes from the TCGA data sets.** Performance is measured by *R*. The predictive performance was assessed across 100 MCCV replicates. BRCA: the breast cancer; CRC: the colon and rectal cancer; COAD: the colon cancer; PAAD: the pancreatic cancer.


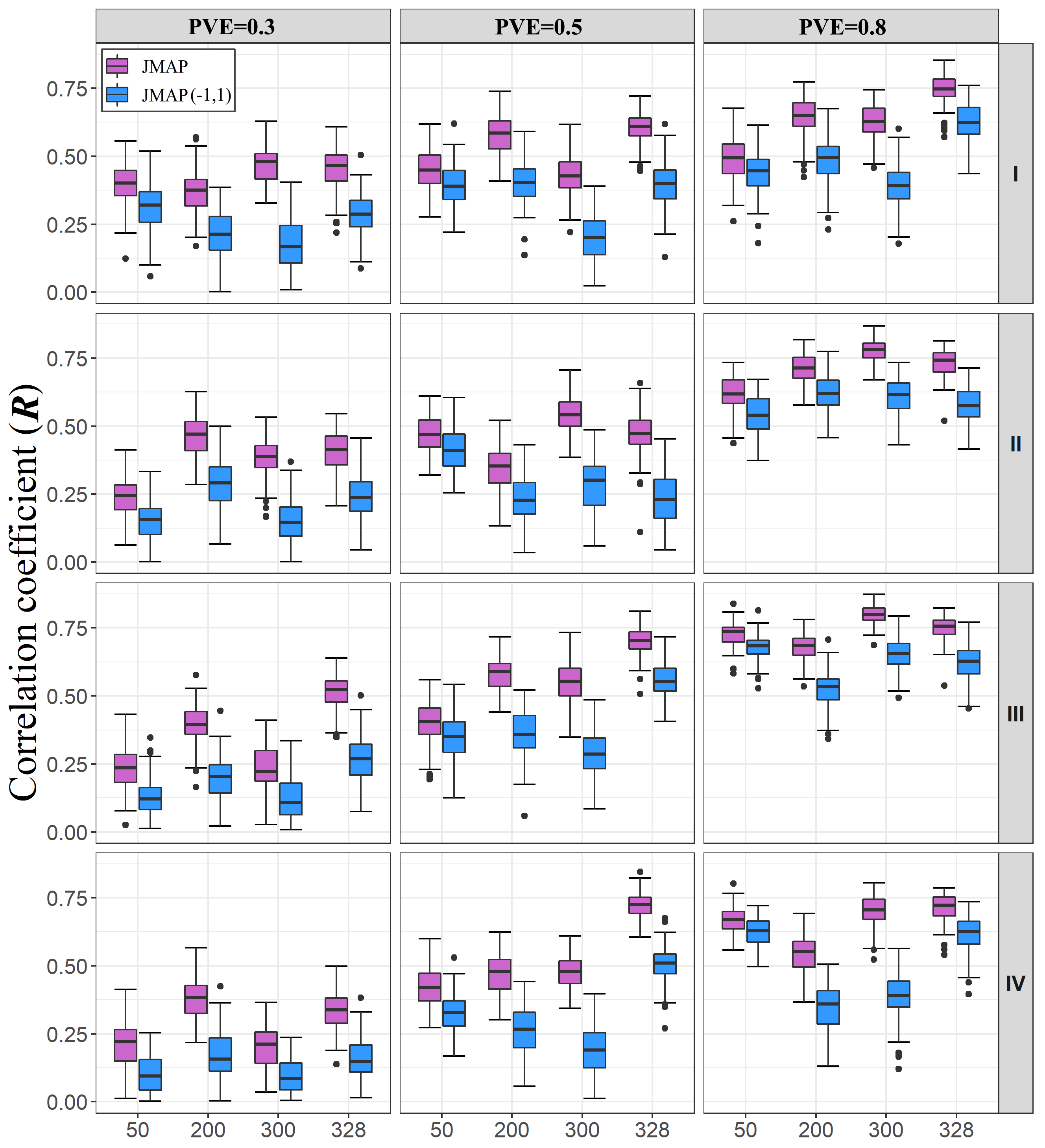


**Fig. S7 Comparison of predictive performance of JMAP and JMAP(-1,1) under various simulation settings.** JMAP represents the method shown in the present study, which assumes the model weights can vary freely between 0 and 1; while JMAP(-1,1) is a variant of JMAP, which further allows the model weights can vary freely between -1 and 1. Performance is measured by *R*; In each setting, five groups with non-zero effect sizes were selected; I represents the settings where all the effect sizes of five groups were all non-zero; II represents the settings where the effect sizes of the first two groups were non-zero, but 50% of the rest three groups were zero; III represents the settings where the effect sizes of the first two groups were non-zero and the proportion of non-zero effect sizes in the last three groups was 80%, 50% or 20%, respectively; IV represents the settings where the proportion of non-zero effect sizes in the five groups was 90%, 70%, 50%, 30% or 10%.The predictive performance was assessed across 100 replicates in each scenario.


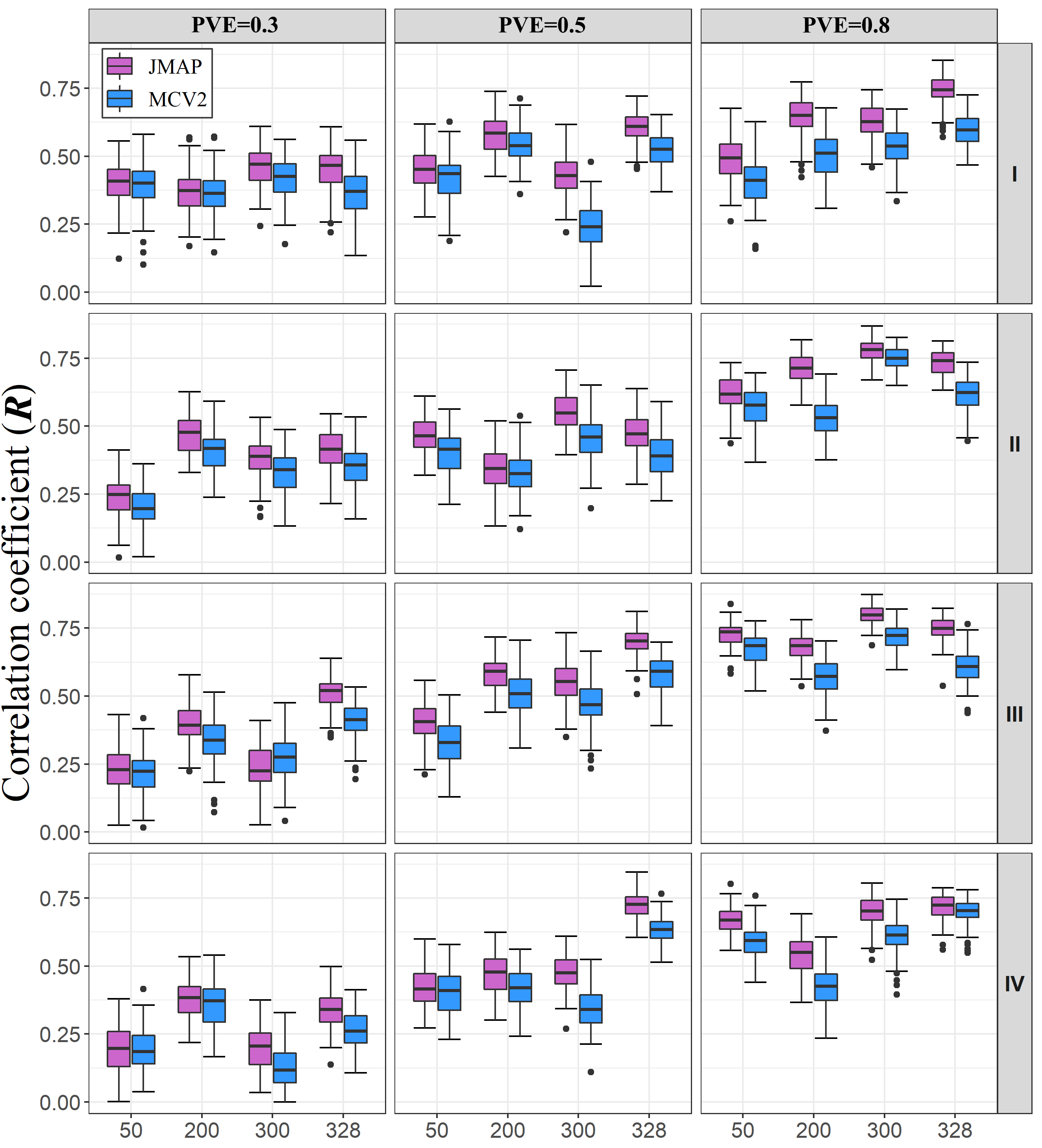


**Fig. S8 Comparison of predictive performance of JMAP and MCV2 under various simulation settings.** JMAP represents the method shown in the present study, which assumes that the candidate models are preassigned and the model weights can vary freely between 0 and 1; MCV2 is the model averaging method descripted in , where the candidate models are constructed based on the marginal correlation magnitude between each predictor and the response. Performance is measured by *R*; In each setting, five groups with non-zero effect sizes were selected; I represents the settings where all the effect sizes of five groups were all non-zero; II represents the settings where the effect sizes of the first two groups were non-zero, but 50% of the rest three groups were zero; III represents the settings where the effect sizes of the first two groups were non-zero and the proportion of non-zero effect sizes in the last three groups was 80%, 50% or 20%, respectively; IV represents the settings where the proportion of non-zero effect sizes in the five groups was 90%, 70%, 50%, 30% or 10%.The predictive performance was assessed across 100 replicates in each scenario.


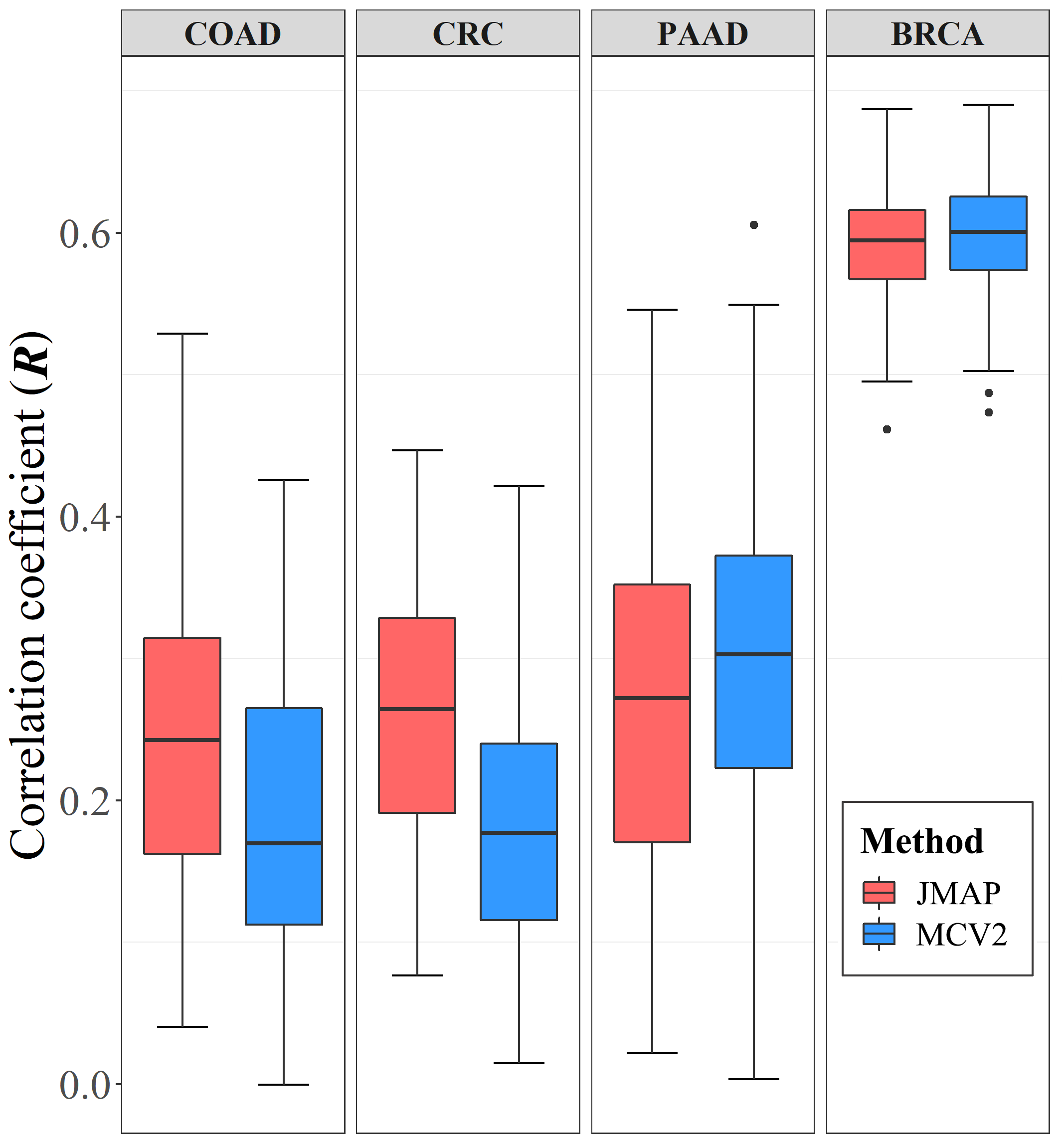


**Fig. S9 Comparison of predictive performance of JMAP and MCV2 for four phenotypes from the TCGA data sets.** JMAP represents the method shown in the present study, which assumes that the candidate models are preassigned and the model weights can vary freely between 0 and 1; MCV2 is the model averaging method descripted in , where the candidate models are constructed based on the marginal correlation magnitude between each predictor and the response. The predictive performance was assessed across 100 MCCV replicates. BRCA: the breast cancer; CRC: the colon and rectal cancer; COAD: the colon cancer; PAAD: the pancreatic cancer.


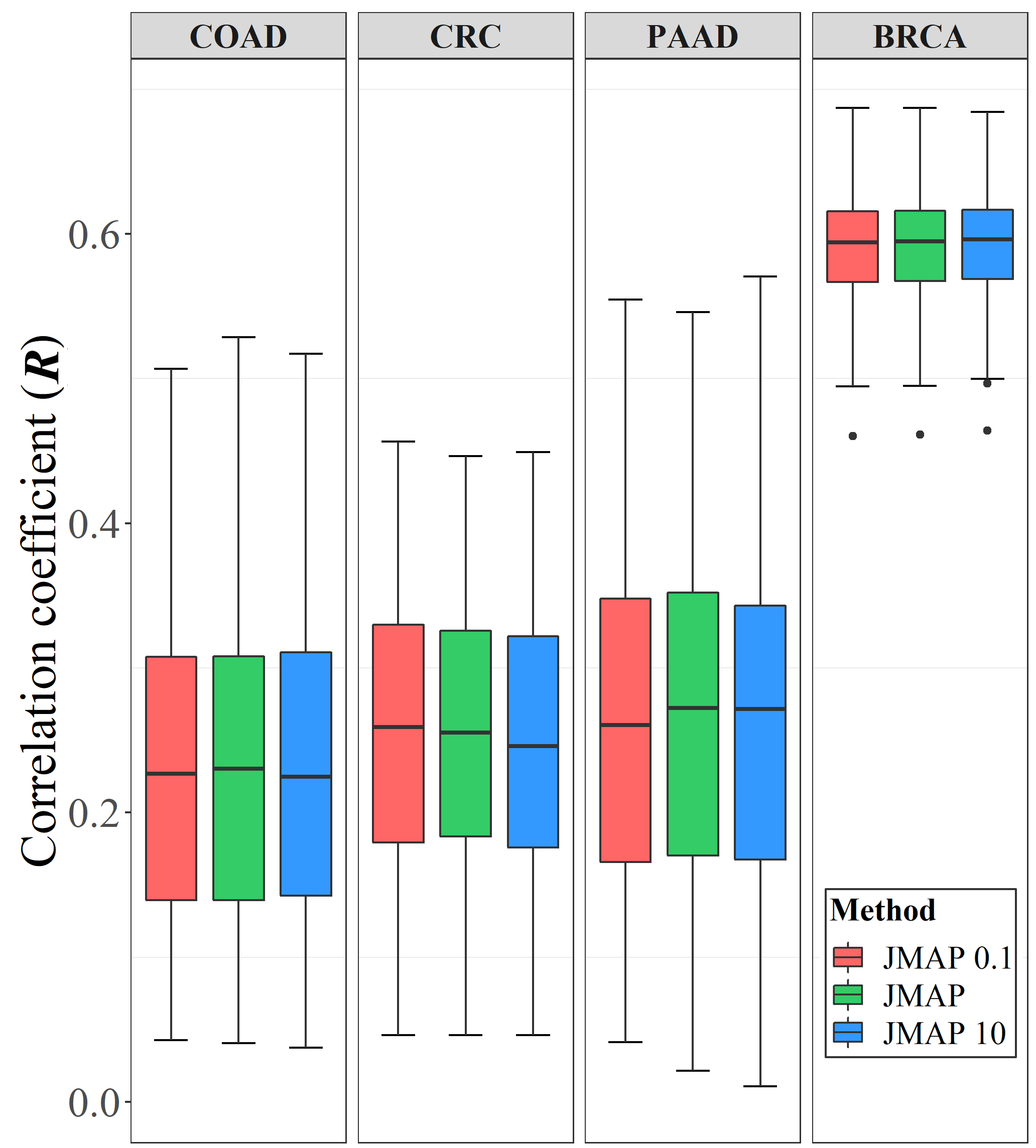


**Fig. S10 Comparison of prediction performance of JMAP with various δ values for four phenotypes in the TCGA datasets when the number of predictors in the candidate model is larger than the sample size.** JMAP 0.1 represents the results when δ was set to 0.1; JMAP represents the results shown in the present study when δ was set to 1, which was also the default option of JAMP when the number of predictors in the candidate model is larger than the sample size; JMAP 10 represents the results when δ was set to 10. BRCA: the breast cancer; CRC: the colon and rectal cancer; COAD: the colon cancer; PAAD: the pancreatic cancer.

**Table S1 Estimated model weights for the true and null candidate models of JMAP in the simulated data sets.**

| Model | groups | PVE=0.3 | | | | |  | PVE=0.5 | | | | |  | PVE=0.8 | | | | |
| --- | --- | --- | --- | --- | --- | --- | --- | --- | --- | --- | --- | --- | --- | --- | --- | --- | --- | --- |
|  |  | prop of  weights=0 | |  | mean ± sd for  non-zero weights | |  | prop of  weights=0 | |  | mean ± sd for  non-zero weights | |  | prop of  weights=0 | |  | mean ± sd for  non-zero weights | |
|  |  | True | null |  | True | null |  | True | null |  | True | null |  | True | null |  | True | null |
| **I** | 50 | 41.6 | 73.6 |  | 0.09±0.06 | 0.06±0.04 |  | 24.2 | 76.4 |  | 0.11±0.07 | 0.06±0.06 |  | 10.4 | 80.3 |  | 0.16±0.07 | 0.05±0.04 |
|  | 200 | 55.0 | 93.4 |  | 0.11±0.07 | 0.07±0.06 |  | 60.8 | 90.7 |  | 0.10±0.07 | 0.07±0.06 |  | 0.0 | 97.2 |  | 0.16±0.07 | 0.05±0.04 |
|  | 300 | 53.6 | 93.7 |  | 0.13±0.09 | 0.07±0.06 |  | 50.2 | 95.1 |  | 0.15±0.15 | 0.07±0.06 |  | 0.0 | 98.8 |  | 0.16±0.07 | 0.05±0.04 |
|  | 328 | 28.2 | 92.8 |  | 0.16±0.11 | 0.09±0.13 |  | 23.0 | 96.3 |  | 0.24±0.22 | 0.07±0.08 |  | 45.8 | 93.1 |  | 0.17±0.1 | 0.10±0.15 |
| **II** | 50 | 28.8 | 71.5 |  | 0.14±0.11 | 0.08±0.12 |  | 29.0 | 71.4 |  | 0.21±0.20 | 0.07±0.07 |  | 14.8 | 77.7 |  | 0.17±0.09 | 0.08±0.14 |
|  | 200 | 71.0 | 91.9 |  | 0.14±0.10 | 0.08±0.11 |  | 22.6 | 92.6 |  | 0.21±0.18 | 0.07±0.07 |  | 0.0 | 97.1 |  | 0.17±0.09 | 0.08±0.13 |
|  | 300 | 31.8 | 94.8 |  | 0.15±0.10 | 0.08±0.10 |  | 19.6 | 96.4 |  | 0.22±0.17 | 0.06±0.07 |  | 21.2 | 97.0 |  | 0.21±0.12 | 0.08±0.12 |
|  | 328 | 81.2 | 93.0 |  | 0.14±0.10 | 0.08±0.11 |  | 12.0 | 93.1 |  | 0.22±0.16 | 0.07±0.09 |  | 41.6 | 93.3 |  | 0.22±0.13 | 0.08±0.13 |
| **III** | 50 | 76.6 | 74.0 |  | 0.14±0.10 | 0.08±0.11 |  | 56.0 | 67.4 |  | 0.21±0.16 | 0.07±0.09 |  | 20.2 | 78.3 |  | 0.21±0.12 | 0.08±0.13 |
|  | 200 | 55.6 | 91.0 |  | 0.14±0.10 | 0.08±0.10 |  | 20.0 | 95.5 |  | 0.21±0.15 | 0.07±0.09 |  | 14.4 | 97.5 |  | 0.23±0.13 | 0.08±0.13 |
|  | 300 | 27.8 | 94.7 |  | 0.15±0.11 | 0.08±0.10 |  | 21.4 | 97.1 |  | 0.23±0.17 | 0.07±0.09 |  | 24.6 | 96.3 |  | 0.25±0.15 | 0.07±0.12 |
|  | 328 | 52.2 | 92.0 |  | 0.15±0.11 | 0.08±0.10 |  | 32.0 | 92.3 |  | 0.22±0.16 | 0.07±0.09 |  | 23.8 | 94.5 |  | 0.24±0.15 | 0.07±0.12 |
| **IV** | 50 | 46.6 | 76.0 |  | 0.14±0.11 | 0.08±0.10 |  | 37.2 | 76.4 |  | 0.21±0.16 | 0.07±0.09 |  | 14.8 | 82.1 |  | 0.23±0.14 | 0.07±0.12 |
|  | 200 | 54.0 | 91.9 |  | 0.14±0.10 | 0.08±0.10 |  | 35.2 | 93.4 |  | 0.21±0.16 | 0.07±0.09 |  | 40.0 | 97.5 |  | 0.25±0.15 | 0.07±0.12 |
|  | 300 | 66.0 | 94.2 |  | 0.14±0.11 | 0.08±0.10 |  | 46.4 | 97.3 |  | 0.22±0.16 | 0.07±0.09 |  | 39.2 | 97.1 |  | 0.26±0.15 | 0.07±0.12 |
|  | 328 | 66.2 | 91.9 |  | 0.14±0.10 | 0.08±0.11 |  | 24.2 | 95.9 |  | 0.22±0.16 | 0.07±0.09 |  | 58.4 | 94.7 |  | 0.25±0.15 | 0.07±0.11 |

**Note**: True is the candidate model that has non-zero effect sizes; null is the candidate model that has zero effect sizes; and prop of weights=0 denotes the proportion of model weights that were estimated to be zero in JMAP.

## Reference

1. Hansen BE, Racine JS: **Jackknife model averaging**. *Journal of Econometrics* 2012, **167**(1):38-46.

2. Ando T, Li K-C: **A model-averaging approach for high-dimensional regression**. *Journal of the American Statistical Association* 2014, **109**(505):254-265.

3. Li K-C: **Asymptotic optimality for Cp, CL, cross-validation and generalized cross-validation: discrete index set**. *The Annals of Statistics* 1987:958-975.
